# Compassion Focused Approaches to Working With Distressing Voices

**DOI:** 10.3389/fpsyg.2019.00152

**Published:** 2019-02-01

**Authors:** Charles Heriot-Maitland, Simon McCarthy-Jones, Eleanor Longden, Paul Gilbert

**Affiliations:** ^1^Glasgow Mental Health Research Facility, Institute of Health and Wellbeing, University of Glasgow, Glasgow, United Kingdom; ^2^Department of Psychology, Institute of Psychiatry, Psychology and Neuroscience, King’s College London, London, United Kingdom; ^3^Department of Psychiatry, Trinity College Dublin, Dublin, Ireland; ^4^Psychosis Research Unit, Greater Manchester Mental Health NHS Foundation Trust, Manchester, United Kingdom; ^5^Centre for Compassion Research and Training, University of Derby, Derby, United Kingdom

**Keywords:** compassion, CFT, auditory verbal hallucinations, voice-hearing, shame, trauma

## Abstract

This paper presents an outline of voice-hearing phenomenology in the context of evolutionary mechanisms for self- and social- monitoring. Special attention is given to evolved systems for monitoring dominant-subordinate social roles and relationships. These provide information relating to the interpersonal motivation of others, such as neutral, friendly or hostile, and thus the interpersonal threat, versus safe, social location. Individuals who perceive themselves as subordinate and dominants as hostile are highly vigilant to down-rank threat and use submissive displays and social spacing as basic defenses. We suggest these defense mechanisms are especially attuned in some individuals with voices, in which this fearful-subordinate – hostile-dominant relationship is played out. Given the evolved motivational system in which voice-hearers can be trapped, one therapeutic solution is to help them switch into different motivational systems, particularly those linked to social caring and support, rather than hostile competition. Compassion focused therapy (CFT) seeks to produce such motivational shifts. Compassion focused therapy aims to help voice-hearers, (i) notice their threat-based (dominant-subordinate) motivational systems when they arise, (ii) understand their function in the context of their lives, and (iii) shift into different motivational patterns that are orientated around safeness and compassion. Voice-hearers are supported to engage with biopsychosocial components of compassionate mind training, which are briefly summarized, and to cultivate an embodied sense of a compassionate self-identity. They are invited to consider, and practice, how they might wish to relate to themselves, their voices, and other people, from the position of their compassionate self. This paper proposes, in line with the broader science of compassion and CFT, that repeated practice of creating internal patterns of safeness and compassion can provide an optimum biopsychosocial environment for affect-regulation, emotional conflict-resolution, and therapeutic change. Examples of specific therapeutic techniques, such as chair-work and talking with voices, are described to illustrate how these might be incorporated in one-to-one sessions of CFT.

## Introduction

The experience of hearing voices that other people cannot hear, variously referred to as “hearing voices,” “voice-hearing” or “auditory verbal hallucinations” has been reported for millennia (McCarthy-Jones, 2012). Extended voice-hearing experiences are reported by around 2.5% of the general population without a subsequent need for psychiatric care (McGrath et al., 2015). However, when voices are frequent, uncontrollable, and have negative content, they are likely to result in distress and problems with functioning (Daalman et al., 2011). Diagnoses that people distressed and impaired by voice-hearing may receive, depending on the other experiences that co-occur with the voices, include schizophrenia, bipolar disorder, post-traumatic stress disorder, and borderline personality disorder (Larøi, 2012).

Contemporary neurocognitive models of voice-hearing have treated the question to be answered as “why people are hearing a voice in the absence of an external stimulus”; a query that is largely driven by formal definitions of the experience in purely perceptual terms (David, 2004). This has led research to focus on the auditory system and, correspondingly, models of voice-hearing as an aberrance of perception. However, a more recent conception of voice-hearing proposes it to be a hallucinated social communication (Bell, 2013). This offers the potential for a subtly different explanatory path to be followed, which starts with the question of why and how we monitor for social communications.

### Evolution and Communicative Monitoring

There is general agreement that many of our basic motivational and emotional systems are deeply rooted in our mammalian heritage (Buss, 2014; Gilbert, 2016). These motivational systems serve essential reproductive and survival functions and, importantly, all require stimulus detection and behavioral repertoires to successfully pursue these motives. This can be seen in relation to feeding, seeking sexual partners or avoiding harms such as predators. In addition to the basic motives brains evolved with, there are also various processing systems that monitor stimulus configurations in terms of their “meaning.” These seek answers to questions such as, “is this sensory stimulus an indicator of food or poison?,” “is this sound or smell indicative of a possible predator?,” “is this sound a distress call from offspring?,” and “is that display by conspecifics offering opportunities for reproduction?” In group-living animals, monitoring systems are specifically orientated to social location; that is, awareness of the physical presence (nearness or distance) and intentions of others. For example, in the attachment system, a parent remains vigilant to the distress signals, and the distance s/he is, from her offspring (Bowlby, 1973).

Of potential relevance to voice-hearing is the social monitoring system of conspecifics in relation to dominant-subordinate behavior (Gilbert, 2000). Dominants and subordinates carefully monitor each other’s presence, whereabouts, and behavior. For example, dominant primates will monitor the behavior of those subordinate to them to ensure they are not inappropriately accessing or attempting to access resources, and will offer threat displays if they are. To those to whom they are subordinate, they will monitor the intention and potential for stimulating aggressive intent. This leads to what Chance (1967) has termed “group based attention structure,” which can be fine-tuned to the social location of potentially helpful or hostile others. However, dominant primates can be unpredictable and launch unexpected attacks on subordinates; partly as a tactic to obtain subordinate attention as well as to maintain levels of stress/fear (Gilbert and McGuire, 1998).

An understanding of voice-hearing can hence begin with the recognition that over many millions of years, mammals evolved attentional mechanisms that are primed for social location and the friendliness or hostility of others with whom one might be in close proximity. The roots for voice-hearing may lie in the evolution of the way language has become recruited into, and utilized by, a range of self-monitoring systems that are pursuing innate motivational biopsychosocial goals. To be specific, an important part of the social brain hypothesis (Dunbar, 2016) is the way in which human brains have evolved to represent relationships. As humans, we can represent in our minds the voices of each other’s thoughts and the possible threat-thoughts that others might have. We can imagine the threat-relevant conversations someone might have about us, which of course requires language, but also the representation of the speech of others. This is something that we all do, and there is a clear evolutionary advantage in this; for example, we can work out that if we say or do X, how another person might think about us and react. We can run mental simulations in our mind and organize our behavior according to our predictions. If the threat system is highly dominant, then it’s going to be those conversations which will become more prominent. It may be that with voice-hearers, there is a differential experience in this element of mentally representing the voices of others. As will be explored in the next section, this may be linked to dissociative processes in response to, e.g., trauma and/or shame.

### Threat, Shame, and Self-Criticism

There is a strong association between voice-hearing and childhood traumas (Read et al., 2003; Janssen et al., 2004; Bentall et al., 2012; Varese et al., 2012; Kelleher et al., 2013; Longden et al., 2016) meaning that many voice-hearers have, as children, experienced subordination to a dominant other (Romme et al., 2009). This is likely to have led to the development of highly sensitized threat-monitoring systems, especially for threats from dominant powerful others who may have malevolent intent. However, importantly, it is not just high threat activation alone that is sufficient to lead to voices (many people experience trauma without developing voices), and therefore consideration of an individual’s threat-regulatory resources is also crucial. Hence where there is high threat activation (e.g., from interpersonal traumatic experience) *combined with* low regulatory capacity in the soothing system (e.g., from affiliative/attachment experiences), there is more likelihood of such heightened monitoring of threat-intentions from others. This state of affairs offers an explanation for voice-content, as the majority of voices heard in clinical populations involve threats (Nayani and David, 1996;McCarthy-Jones et al., 2014). It also offers an explanation for a common form of relating to voices, which involves feelings of being in relation to a more powerful figure (Chadwick et al., 2000; Hayward, 2003), as subordination to one’s voices is closely linked to experiences of subordination and marginalization in other social relationships (Birchwood et al., 2000; Gilbert et al., 2001; Birchwood et al., 2004). Importantly too, it offers a number of potential explanations for the etiology of the voices. Hypervigilance for social threat has been proposed to be a key mechanism involved in at least some voice-hearing experiences (Dodgson and Gordon, 2009; Garwood et al., 2015). Whist this threat may be physical (Dodgson and Gordon, 2009) it may also be a threat to one’s social status. Given that the latter form of threat is often signaled by shame, referred to as the “affect of inferiority” (Kaufman, 1989) (p. 16), this suggests a potential role for shame in the etiology of some voice-hearing (McCarthy-Jones, 2017b).

Shame has two components. One is linked to what is called external shame, which has an external focus for attention, thinking and coping, whereas with internal (or internalized) shame, the attention is focused in on the self, with high levels of self-criticism. External shame involves individuals monitoring, experiencing, or imagining that they are the focus of other people’s criticism, rejections and desires to exclude, marginalize or even persecute them. In other words, rejecting and malevolent intent by others. External shame is hence the experience of becoming an unattractive and undesirable agent in the mind of others (Gilbert, 1998). In contrast, internalized shame, is linked to carrying those evaluations into the sense of self. Internal shame requires that there has to be some perception of self as actually “unattractive” - not just a failure to reach a standard (Gilbert, 1992, 2002); that is to say it is closeness to an undesired and unattractive self rather than distance from a desired self that is at issue (Ogilvie, 1987). These observations are important because most malevolent voices attack individuals as if they are in some way unattractive, undesirable, or an object of derision or disgust – a sort of “anti-ideal” to use De Rivera and Mascolo language – or undesired self (Ogilvie, 1987). It is now known that many human competitive interactions rarely involve outright aggression and much more communications and degradation of status; that is via shaming. Men and women denigrate and shame each other slightly differently, often in domains of sexual attractiveness or competency (Buss and Dedden, 2016). Legg and Gilbert (2006) found evidence for this in that some voice-hearers experienced the voices giving sexual taunts, such as “you are a slut, ugly, a pervert, smelly disgusting, unattractive.” What is less clear is whether before individuals had voices, they had these anxieties about themselves, possibly even at the unconscious level; i.e., whether the voice is articulating one of their internal fears. In addition to insults, voices behave like hostile-dominant individuals, often showing commands and threats. A comparison of the physiological consequences of being trapped by a hostile-dominant who is regularly putting one down reveals this to be not dissimilar from an internal stream of thinking that is hostile and putting oneself down. In fact, self-criticism operates through similar neurophysiological systems as being criticized by others (Longe et al., 2010). Rather than thinking about voice-hearing in terms of some abnormality in brain chemistry, it may represent the activation of evolved functions for social threat monitoring.

The basic proposition then is that humans evolved with a range of different motivational potentials that organize attention, thinking, behavior and physiological systems quite differently. When we are orientated for sexual behavior, we are in a very different mind state than when we are orientated to avoid threat or for eating behavior. One of the big social motivational systems is linked to social competition, and in particular social rank in the context of social hierarchies that regulate social threat. For individuals who have experienced threat from powerful others, their social threat detection systems are choreographed in the brain in such a way that they can be easily activated, attentionally tuned, and can generate intrusive fears (“you are ugly and a pervert”). Common to many voice-hearers is the permeability of the self, such that these feared aspects of the self cannot be kept secret - others can “know about them” and “know what they are.” This is the fear of detection by a dominant.

Such considerations also point to mediating relationships between shame and voice-hearing. One likely mechanism is dissociation. This is a common way in which people may try to deal with shame (Talbot et al., 2004; Dorahy and Clearwater, 2012). In survivors of trauma, levels of shame are positively associated with levels of dissociation (Talbot et al., 2004). Dissociation additionally has a strong association with voice-hearing [see Longden et al. (2012) and Pilton et al. (2015) for a review], potentially due to its eliciting of cognitive intrusions (Dorahy et al., 2017). With prevailing conceptual and clinical links to psychosis, the case has been made that all voice-hearing can essentially be understood as dissociative in nature (Moskowitz and Corstens, 2008). In this respect, Van der Hart et al. (2006) structural model of dissociation proposes that trauma exposure may divide the personality into systems that are focused on daily life and functioning, and systems that are threat-protective and fixated on survival (comprising defense subsystems such as apprehend, fight, flight, freeze, submit). Within such a framework, voice-hearing can therefore be conceived as an experience of ‘disowned’ threat-based representations of the self (or self-other relationships), intruding upon functioning-focused parts of the personality or self. Hence why voices are experienced as cognitively and perceptually detached from autobiographical experience (Dorahy and Palmer, 2015).

As noted by Longden et al. (2018a), a dissociative framework for voice-hearing accounts for several aspects of the experience that cannot be wholly understood using cognitive/perceptual models alone; including for example, why voices may often speak in the second or third person, their semantic and syntactic complexity and varied personifications (e.g., different ages, genders and response styles) and the type of intricate interpersonal dynamics described previously, wherein voices relate to the hearer in ways that reflect broader patterns of social relating.

*“Specifically, moving beyond the cognitive paradigm of the mind as a computer, mental processes can be understood as a product of social influences through which evolutionary processes (determined by culture) lead to internalized values, beliefs, and behavioral patterns from caregivers and other significant figures. In turn, this internalization of cultural patterns is expressed and represented in internal dialogic processes that embody the person’s relationship with their environment. By extension, this concept invites an understanding of the self, not as a unitary or centralized entity that governs the life of the person, but rather as a dynamic, complex and heterogeneous experience that is formed by biographical influences and which allows the interplay of different self-positions that have an adaptive role for the person. As such, this framework expands the view of…[voice-hearing] as merely hallucinations and perceptual aberrations, and instead focuses on states of consciousness reflecting different dissociated positions of the self; positions which, in the majority of cases, are experienced as subjectively real and are in conflict with one another”* (Longden et al., 2018a).

In turn, conceptualizing distressing voices as representations of one’s sense of self suggests a strong clinical rationale for finding ways of engaging with them that can promote more peaceful, positive interactions between hearer and voice, reduction of dissociative divisions, and recognition of their “protective” function by drawing attention to unresolved emotional conflicts (Corstens et al., 2012; Moskowitz et al., 2017; Mosquera and Ross, 2017). Jacqui Dillon, the Chair of the United Kingdom Hearing Voices Network, expresses the imperative in the following way: “Each voice is an echo of the person’s experience so an attitude of curiosity, understanding and compassion toward all voices is the best stance as it will encourage and support internal communication and, ultimately, self-acceptance” (Dillon, 2013).

## A Compassion Focused Therapy Approach to Voice-Hearing

Social competition and hierarchical social organization is only one of a number of social motivational systems. Another is supportive and caring behavior, and when this motivational system is orientated it organizes attention, behavior, thoughts and physiology in very different ways. Hence one therapeutic maneuver would be therefore to switch individuals from a threat-based hierarchical competitive motivational system into a care focused one. This is not likely to be easy because these switches can involve a number of fears, blocks, and resistances; part of which is, of course, mistrust. If we come to locate some of the difficulties that people with distressing voices experience as sensitized competitive motivational and threat-protective processing systems, then one therapeutic avenue is to begin to help individuals switch out of those particular motivational systems and to develop new ways of emotional regulation – ones that are not simply threat based – and begin to create a sense of the secure base and safe haven. Compassion focused therapy (CFT) attempts to do this by showing individuals how they can switch to different motivational orientations and learn different emotion regulation skills. To create the insider motivational impetus for this work, CFT uses a lot of psychoeducation about the evolved nature of the mind. Therefore, in the case of people who are voice-hearers, it can be very useful to invite them to think about some of the processes discussed above. For example, the fact that all mammals, including and especially humans, have monitoring systems that are designed to try and detect potential social threats; and that sometimes these systems can become highly sensitized, particularly if we’ve been in traumatizing situations. We can also discuss the fact that *this is not our fault* (which in itself can have important de-shaming potential) and that it is very easy for us to create an exaggerated sense of shame, partly because of the profound importance of shame, evolutionarily, for the regulation of social behavior. Crucially, however, motivational interviewing techniques can be used to orientate people to the idea that they could begin to notice these dominant-subordinate motivating and monitoring systems and, when they arise, to shift to a compassion system.

The reason for doing this is that compassion stimulates a completely different set of neurophysiological systems and has a profound effect on threat processing. So the focus of the therapy is less on preventing actual voice-hearing and more on helping individuals switch motivational systems from which those voices are arising. So the essence of the therapy is to set up a psychoeducation framework and then provide breathing, postural and verbal tone training (described in more detail below). Individuals then practice engaging and imagining themselves at their compassionate best, rooting themselves in compassionate courage and wisdom, and beginning to think about how they would wish to engage with their inner voices from the position of their compassionate self. That constant practice of inhabiting and creating the inner patterns of compassion becomes the source of change and growth. Indeed, in a qualitative study, Waite et al. (2015) found that, in recovery from psychosis, self-criticism and self-compassion were linked to two different cycles of outcome: Shame based self-criticism was associated with increasing distress over psychotic experiences, whereas self-compassion was associated with empowerment and growth. There is also evidence that helping people to generate compassion motivation and emotion through practices such as compassionate identity or compassionate self-training may be therapeutically beneficial (Braehler et al., 2013).

### Creating Biopsychosocial Conditions to Facilitate Engagement With Threat-Based Emotion and Processes

Compassion focused therapy aims to help people develop their capacity for affiliative relating (with self, voices, and other people) through the cultivation of compassion at each of the bio-, psycho-, and social levels. The rationale for targeting these processes and mechanisms in CFT is based on extensive research on the physiology of emotion regulation through social and affiliative experience (reviewed by Hostinar et al., 2014), as well as evidence for how compassion exercises/practices can influence different physiological processes that are important for emotional wellbeing (Pace et al., 2009; Mascaro et al., 2013; Weng et al., 2013). By helping to foster these conditions within a person’s body, mind, and social environment, CFT aims to give the voice-hearer a better chance of engaging with, understanding, and integrating their threat-based emotions and experiences.

#### Biological Aspects: The Physiology of Social Safeness

At the biological level, compassion is supported by bodily experiences of social safeness and groundedness. To inform interventions, CFT has sought to understand the neurophysiological underpinnings of these processes, particularly their links to activity of the vagus nerve and heart rate variability (HRV) (Kirby et al., 2017). Indeed, HRV has been increasingly adopted in CFT research as an important bio-marker measurement of safeness physiology. This is reflected in the current CFT for psychosis research underway in the United Kingdom, which includes HRV measures at five points over the 6 to 8-month course of therapy (ClinicalTrials.gov identifier: NCT02733575).

In the same way that bodily experience of safeness will support the types of mental states that are conducive to compassion (e.g., mentalising, empathy, etc.), compassionate intentions and actions can in turn enhance experiences of social safeness, affiliation, and connection. Hence, safeness physiology and compassion mentality are mutually supportive.

It is for this reason that CFT interventions for voice-hearers typically start with a focus on establishing social safeness and groundedness. These provide the foundations for subsequent relational work with self, emotions, voices, and other people. A range of practices are utilized in CFT to directly stimulate safeness physiology; e.g., soothing breathing, grounding, body posture, facial expression and verbal tone. Some brief descriptions of these are summarized below, and for more a detailed exploration of these techniques see Kirby (2017).

##### Grounding and body posture

This involves practicing certain body postures and movements to support the desired patterns of physiology and mentality. For example, adopting more upright and expansive postures are more likely to send signals of calm composure and confidence to the mind, in contrast to more inward and tighter body postures, which are more likely to signal anxiety, threat, and danger.

##### Soothing breathing practice

This involves practicing slowing down the rhythm of breathing, whilst paying mindful attention to sensations of slowing in the body. The practice of slow, even, smooth breathing can bring feelings of calmness/settling and groundedness, which can be helpful for steadying and anchoring when dealing with threat-based emotions and experiences.

##### Facial expression and voice tone

This involves practicing different facial expressions and voice tones that reflect and support compassionate intentions and motives. There are often certain “tones” that come with threat-based self-monitoring (thoughts and voices). As these are driven by the threat system, i.e., serving interests of protection and survival, these tones can be loud, critical and hostile – effective for salience and attention-grabbing. Practicing self-monitoring with friendlier, warmer, and more supportive tones can help shift body and mind into more compassionate patterns.

#### Social Aspects: Interpersonal Safeness and Affiliation

The finding that dominant-subordinate social relationships are often mirrored in people’s relationships with their voices (Birchwood et al., 2000; Birchwood et al., 2004) highlights a clear need to extend interventions into the social sphere. Furthermore, access to safe and validating social experiences is found to be a protective factor in clinical versus non-clinical outcomes for those with anomalous experiences (Heriot-Maitland et al., 2012; Brett et al., 2014). In CFT, it is therefore important to consider the interpersonal environment, and the role of relationships, interactions and social contexts in supporting the patterns and processes outlined above. Drawing on roots in Attachment Theory (Bowlby, 1973), CFT emphasizes the links between social affiliative experience and the “soothing system”; the term used in CFT to refer to the body’s natural systems of (parasympathetic) slowing, calming, and settling. As Bowlby observed in infant-carer interactions, the soothing system is highly sensitive to signals of inter-personal safeness, kindness and care, and has an important role in regulating the (sympathetic) arousal of the threat system. These social attachment mechanisms have been further examined and elaborated in the field of evolutionary neuroscience (Porges, 2007).

Important for CFT, however, is recognizing when experiences of social attachment and affiliation may *not* activate soothing/safeness system, but rather the threat system – as may be the case for many voice-hearers. This can be due to attachment–threat conditioning through early interpersonal experiences, particularly where childhood experiences with caregivers have been aversive or inadequate. For these individuals, it will understandably be harder to access the soothing/safeness effects of caring experiences, alongside having reduced resource for threat regulation. In CFT, the aim would be to increase a person’s capacity for affiliative relating by gradual exposure to affiliative experiences, with simultaneous exposure and de-sensitizing of the elicited threat emotions. Recruiting the physiological grounding, posture and breathing techniques outlined above is an important way of supporting this challenging exposure work.

#### Psychological Aspects: Compassion Mentality

At the psychological level, CFT aims to help voice-hearers tap into compassionate motives and mentalities by practicing certain patterns of cognition, planning, memory, and imagery. Difficulties with mentalising have been highlighted as particularly relevant among people with psychosis-related diagnoses (Liotti and Gumley, 2008), and so cognitive work in CFT will typically focus on exercises that develop mentalising capacity. For example, mindfulness skills are often practiced in CFT on the grounds that mindfulness can not only help people to improve their attentional skills as a tool and vehicle toward compassion, but also develop (mentalised) awareness of the contents and patterns of their mind. Awareness of these mental states, loops and patterns, particularly the threat-based self-monitoring and social-monitoring described above, will enable people to develop a relationship *to* them, rather than be caught up *within* them. In CFT, the psychoeducation about evolved “tricky brains” helps to ensure that this relationship is one of understanding and compassion; for example, recognizing that *it’s not our fault* that these processes occur in our minds due to our evolutionary threat protection and survival needs. This helps to reduce self-criticism and shame, which, as we have already seen, may be key in both the etiology and the emotional consequences of voice-hearing.

Memory and imagery techniques are used in CFT to support the development and training of compassionate psychologies. For example, people are invited to bring to mind remembered and/or imagined compassionate relationships with, e.g., safe places, compassionate others, and themselves. CFT will always seek to proceed at a pace that allows time and space to mindfully notice the bodily expressions of these memories and images. This helps the therapist and client to notice and address fears, blocks and resistances as they arise (e.g., see fears of compassion scales (Gilbert et al., 2011), and aids the process of compassion exposure and desensitization.

### Cultivating and Deepening the Compassionate Self

In CFT, each of the bio-, the psycho- and the social- components of compassionate mind training (outlined above) are brought together as a self-identity called the compassionate self. Essentially the compassionate self operates as a holder for all the various skills, practices, postures, etc that are trained; an inner sense of having the qualities required to bring compassion to one’s self-to-self and other relationships. In the case of voice-hearers, the compassionate self is an inner sense of the qualities and skills that are required to bring greater understanding, care, and peace into the relationship with one’s voices.

Evidence from Matos et al. (2017) has shown that the more a person succeeds in embodying the compassionate mind training in their everyday lives (i.e., via a compassionate self), the more this improves their perceived feelings of safeness and their compassionate relating. As such, cultivating and deepening a compassionate self can be regarded as important preparatory work for compassionate engagement with voices, and indeed with any other threat-based emotion or conflict that may be causing problems. The idea is that it leads the person to a point of greater readiness to do what’s required in therapy. Whatever the therapy goals may be (e.g., processing a trauma memory, managing dissociative states, overcoming a social fear, finding a job, etc.), the compassionate self becomes the place to both *come from* and *go back to* when engaging with the required emotional work. In therapy, the compassionate self also takes the role of an internal supportive presence and guide throughout.

## Compassion for Voices, Multiple Selves, and Emotional Parts

In this section, we focus on direct emotional work with multiple selves as a demonstration of how compassionate self can be used in CFT for people who hear voices. In CFT, this is often referred to as *directing the compassionate self* or *putting the compassionate self to work*.

The therapeutic process of relating to emotions through multiple “selves” or “parts” is not unique to CFT. There are similar constructs and processes outlined in different approaches. For example, in Jungian analysis, an individual is helped to communicate with various parts of their psyche, the *archetypes* (Jung, 1919), so that these can be integrated in consciousness. Similarly, Voice Dialogue, developed by Stone and Stone (2011), offers a framework to help people understand their many parts, some of which are very well known to them, and others which are more distant or disowned. Voice Dialogue encourages people to communicate with their many selves to discover what each wants and needs. More recently, Greenberg has described chair work techniques in Emotion-Focused Therapy (Greenberg and Watson, 2006), which is similarly a way to help access and relate to different emotional parts. What distinguishes CFT from these other “relational” therapies is that the emotional work is conducted from the compassionate self; i.e., the self-identity or part that has been specifically cultivated and trained to be able to compassionately engage with distress. In attachment terms, the compassionate self provides an inner *secure base* and *safe haven*, from which the person can develop strength and courage to confidently explore the emotional world of their threat system.

Identifying where to direct the compassionate self is a collaborative process with the person who hears voices. Some people may wish to initiate dialogs between their compassionate self and their voices, with the aim of understanding the emotional function/meaning of voices, and developing a more peaceful, harmonious relationship with them. For others, there may be a preference to dialog with emotional selves (e.g., angry self, anxious self, or self-critic) that have been identified and conceived within the therapy sessions, and may therefore feel a safer route (initially, at least) toward accessing emotion. For those who do directly dialog with voices, it may be important to seek permission from the voices first, particularly because many voices will have a role of protecting hurt or vulnerable parts (Corstens et al., 2012). So unless the voice feels reassured, respected, and safe, it would make sense why it might try to (protectively) block any process that potentially brings closeness or exposure to a perceived vulnerability. The compassionate self can understand this defensive process, as well as its origins in the threat-protection system, and can therefore meet a voice with validation and sensitivity. An example is given in the short YouTube film, Compassion for Voices (Cultural Institute at King’s, 2015), where the compassionate self, speaking to a critical voice, says:

“I want to understand you. I want to help you feel safe… I know you’re trying to help me. Thank you. Thank you for reminding me that I get scared. You’re right, I do.” (3:02 mins)

Here the compassionate self is calmly and respectfully validating the voice’s protective role, whilst also identifying links between the voice and the emotions of the threat system behind the voice (i.e., fear of leaving the house). In CFT, this kind of dialog might be role-played out in the therapy room using different chairs, which can help the person to connect with an embodied sense of these emotional and motivational systems. So, for example, the voice-hearer might be invited to sit in one chair representing the critical voice, and another chair to represent the compassionate self. When shifting between chairs, the person has time and space to feel their way into each role. So if the voice comes with feelings of frustration, anger or contempt, then the individual would be invited to gradually connect with the associated postures, attitudes and mindset. Similarly with the compassionate self, a shift into this chair will involve plenty of time and space for the person to connect with soothing breathing, groundedness, and the particular qualities required to respond compassionately to this angry voice.

One of the important goals of compassionate dialog might be to understand the nature of the emotion *sitting behind* the voice; i.e., what’s driving the voice; what feeling/experience is the voice protecting? What feeling is there but not yet safe enough to access? This information might be elicited through explorative questioning, for example asking the voice: “Why are you angry?” “What would happen if you stopped or couldn’t be angry?” “What would be your concern then?” Sometimes this kind of questioning may not even be necessary, because voices may respond and open to the encounter (alone) of a safe and compassionate presence. So, for example, the experience of being listened to and validated (e.g., “that sounds really tough for you”; “I can see why you’re frustrated”; “that makes a lot of sense” etc.) may itself create the required safeness to start naturally eliciting the threat-emotional communication.

Once the vulnerability is identified and linked to its existence in the threat system, then this part might itself have a new chair allocated. For some voice-hearers, this part may be a fearful part that has experienced trauma earlier in life. It may be a part that has been bullied, or experienced social shaming, discrimination, and humiliation. This part can gradually become more a focus or recipient of compassionate intent and care. Importantly, the timing and pace will proceed in communication and negotiation with the voice. One creative use of chair work is to use the space and positioning of chairs in the room to represent where the fearful part sits in relation to the voice, and in relation to the compassionate self. For example, the fearful part chair might start off totally hidden behind the voice chair out of sight but then gradually, with the help and encouragement of the compassionate self, come out into its own space in the room, maybe finding its own voice, reflecting more “ownership” of this emotion.

The compassionate chair work can take many different forms depending on the situation. So for instance, it might be helpful to have a chair for the part of self that receives the criticism from the voice. This can give the person a chance to really connect with the dominant-subordinate roles and ranking mentality in operation. The compassionate self might, in this case, be positioned in more of a reflective role: firstly observing and listening to the emotional conflict being played out before responding to both parts with a wise overview of the threat-based functions, and then mediating these parts toward some resolution or integration. This highlights the flexibility required of the compassionate self, and hence the importance in CFT of continuing to train the range of qualities, attributes and skills throughout therapy. In some circumstances, it may be the more gentle, warm and nurturing qualities of compassion that are required. This might be true, for example, when the flow of compassion is directed to an abused child part. Whereas, in other situations, it might be the stronger, more assertive and courageous qualities that are required; for example, when first intentionally opening to the verbal attacks of a hostile voice, which will necessitate considerable courage and tolerance.

In addition to chair work, the process of bringing compassion to voices and emotional parts in CFT employs other techniques such as imagery and letter writing. Compassionate imagery might involve creating imagined characters to represent voices, focusing on characteristics such as facial expression, size, proximity, voice tone, etc. These visual representations can be helpful in enhancing a voice-hearer’s understanding of different aspects and intentions of their voices. They can also provide opportunities for setting up safe imagined scenarios whereby the compassionate self (or an ideal compassionate other) might encounter the voice to start a conversation. With visualizations, there may also be opportunities for the voice-hearer to modify some of these characteristics over time, for example, as the image of the voice receives compassion, there might a slight settling of posture, or lightening of color, or reduction of volume. In compassionate letter-writing, the voice-hearer will often write from the perspective of their compassionate self to a voice or a part, bringing empathic and wise understanding to how it developed and acknowledging its protective role. For more detailed illustrations of these clinical techniques and processes, see a recent single case study account of CFT for relating to voices (Heriot-Maitland and Russell, 2018).

The interpersonal and behavioral work also forms an important part of CFT. This is where the individual is invited to take the compassionate intentions and actions out in their social world of relationships and interactions, therefore developing both the flow of compassion *to* others and the capacity to receive compassion *from* others. As described previously, creating these wider, real-world opportunities for social safeness and connection is a key relational context in which the CFT approach is built. In this regard, the CFT approach to voice-hearing is very compatible with social network approaches in psychosis such as Open Dialogue (Seikkula et al., 2006), Peer-Supported Open Dialogue (Stockmann et al., 2017), and the Hearing Voices Network approach (Corstens et al., 2014; Longden et al., 2018b).

## Conclusion

We have proposed a framework in which voice-hearing can be understood as an interaction between the brain’s evolved systems for paying attention to, decoding and responding to different communication signals relating to different social roles. These signals may be auditory but can also be visual (e.g., facial expressions). We suggest that some voice-hearers can be particularly cued into communication patterns indicating dominant-subordinate social relationships. When that is the social mentality by which the individual is processing social relationships, it becomes primed to detect information about the controlling, critical, and even attacking intent of others, in contrast to the helpfulness. We have argued that the relationships between voices and voice-hearers can be understood in terms of their alignment to social-rank relationships. We have proposed that voice-hearing involves an internal “playing-out” of both the hostile-dominant and the (reciprocal) threatened-subordinate social roles. In other words, just as some (depressed) individuals can experience anger toward themselves, people who are voice-hearers are experiencing these critical condemning attacking emotions as voices rather than as thoughts or sentiments. So while a depressed person may have the thought, “I’ve let myself down; I’m useless; there is no point in living,” the voice-hearer will hear a voice saying, “you’ve let yourself down; you’re useless; there’s no point in you living.” In other words, there is an internal experience of an attacking, condemning part of self *and* a submissive, beaten down part of self (Gilbert et al., 2001).

A number of testable, research hypotheses emerge from this framework. For example, if the voice-hearing experience does indeed reflect evolved social-rank processes, we would expect to find evidence for voice-hearer’s minds being increasingly orientated toward the dominant, controlling, power of others (and voices), and toward the vulnerable, weak, powerlessness of self. This could be researched through psychological studies into, e.g., biased attentional focus and memory recall, as well as through studies into the neurological and physiological underpinnings of threat system activation. Indeed, evidence already suggests that when individuals experience these hostile dominant voices they also experience the social world as hostile-dominant over them, which is sometimes linked to having been harmed by others (Birchwood et al., 2000, 2004). Based on this framework, we would also hypothesize that voice-hearing experiences would be accompanied by a range of other social-ranking characteristics, such as vulnerability to feelings of defeat, inferiority, rejection, and shame, high sensitivity to social comparison, and that voice-hearing would elicit protective strategies such as submission, depression, and dissociation. In particular, we would hypothesize that the more powerful a voice, and the more subordinate a voice-hearer, the higher the levels of, e.g., depression and dissociation. Again, there is some research evidence already suggesting that this may be the case (Gilbert et al., 2001).

In this paper, we have additionally proposed that these social-rank processes can also be moderated by helping voice-hearers activate a different evolved motivational system, linked to mammalian caring behavior. The signals generated when this motivational system is operated are quite different. This motivational system is more likely to give rise to the experience of a secure base and safe haven, in line with attachment theory, and we have suggested that this motivational switching (from competitive to caring) will bring therapeutic benefit to voice-hearers. This also leads to a number of testable research hypotheses around the moderating effects of secure attachment and affiliative experiences. So, while our framework not only generates hypotheses around how external and internal social-ranking signals (such as stigma, shame, self-criticism, and self-stigma), would accentuate threat-based dissociative processes for voice-hearers, it also generates hypotheses about how external and internal communication signals that indicate caring, supportive, and social safeness experiences (e.g., social validation/connectedness and self-compassion) would attenuate them. Finally, from this framework, we would hypothesize that interventions specifically designed to help people switch from social-rank patterns into caring motivational systems, with affiliative signals and patterns, would be beneficial for voice-hearers, and that this would be evidenced by reducing submission, depression and dissociation. Currently there is early evidence indicating that compassionate mind training can be beneficial to voice-hearers (Mayhew and Gilbert, 2008; Braehler et al., 2013), but further evaluation is needed.

The framework outlined in this paper can not only inform the continuing development of psychological interventions for those distressed by the voices they hear, but can also potentially inform contemporary neurocognitive models of voice-hearing. One prominent neurocognitive model of voice-hearing stresses a role for self-monitoring deficits in their etiology (Waters et al., 2012). Our framework adds crucial context to this model, by specifying what type of information is likely being monitored and why. Our framework can also help inform predictive coding accounts of voice-hearing. Such models conceptualize voice-hearing as being a perception resulting from the overweighting of prior expectations (typically under conditions of uncertainty) at the expense of actual sensory input (Powers et al., 2018). Our framework suggests what specific priors are likely to underpin voice-hearing (presence of social threat). It also suggests that a crucial source of information for revising these priors will come from the body. Developing a bodily sense of security through bodily practices such as breathing and grounding, in addition to cognitive approaches, is likely to be necessary to revise such priors. Finally, our framework shares much in common with the hypervigilance model of voice-hearing, which proposes that voices are a by-product of our perceptual system which has evolved to reduce false negatives in conditions of threat (Dodgson and Gordon, 2009). Our framework extends this model by proposing that the evolved threat in question is likely to be a social threat, and offers a particular method through which to reduce such perceived threats (compassion). Thus, our model can be seen to offer a useful expansion of extant neurocognitive models of voice-hearing.

In terms of informing the development of therapeutic approaches for voice-hearing, our framework can be seen to share some commonalities with other recent therapeutic developments in the field, such as Relating Therapy (Hayward et al., 2017) and Avatar Therapy (Craig et al., 2018; Craig, 2019), which both work with voice-hearers to change their relationship with their voices. As our framework involves understanding of the dominant-subordinate motivating and monitoring systems it suggests a specific remedy to the problems caused by these systems; compassion, which is key to aiding affiliative relating. The focus on mindfulness in Compassion Focussed Therapy is consistent with a trend toward utilizing mindfulness techniques to assist those distressed by voice-hearing (Strauss et al., 2015). However, we suggest approaches could be extended by conceptualizing the learnt skill of mindfulness as a tool and vehicle toward enabling compassion.

Earlier, we also noted the use of memory and imagery techniques in Compassion Focussed Therapy, to support the development and training of compassionate psychologies, which can involve bringing to mind remembered and/or imagined compassionate relationships with others. This bears some resemblance to the competitive memory training (COMET) approach of van der Gaag et al. (2012). COMET identifies core themes of the cognitive-emotional memory network activated by voices (such as “incompetence”), creates personal examples of the positive counter theme (e.g., instances of being competent/successful), and puts these into a scene that the patient has to imagine. The patient is then taught to activate this memory at will, with a view to being able to hear the humiliating messages of the voice and not be emotionally affected by it. An RCT of the COMET approach found that although it did not lessen the voices or their negative content (which was not the aim of the study), patients’ depression did decrease (van der Gaag et al., 2012). This was fully mediated by the increase in self-esteem and the acceptance of voices as psychic phenomena, and partially mediated by the attributed power to the voices and the social ranking of oneself in relation to the voices. We would be interested to know whether a focus on specifically compassionate imagery could alter the negative content of voices. Indeed, whilst Van der Gaag and colleagues argue that interpersonal schemata, such as social rank, may mediate the relation between voice-hearing and distress, our framework proposes these may be involved of the etiology of the voices themselves.

This raises the question about what may be hypothesized about how Compassion Focussed Therapy (CFT) can alter the phenomenology of voice-hearing. As noted above, the aim of such an approach is not to eliminate voices but to make them easier to live with. It can first be hypothesized that CFT would lead to a reduction in the negative affective valence of voice-hearing; it will make nasty voices nicer. Given the lack of evidence that cognitive behavioral therapy can reduce the negative affective valance of voices (McCarthy-Jones, 2017a), and that negative valence is the single largest predictor of whether a voice-hearer will have a clinical diagnosis or not (Daalman et al., 2011), if CFT could do this it would be a major clinical achievement. Indeed, a small case series has already offered some intriguing suggestions that CFT may actually be able to reduce the negative affective valence of people’ voices (Mayhew and Gilbert, 2008). And yet, if voice-hearing is grounded in the activation of threat-based (dominant-subordinate) motivational systems, it could also be proposed that by CFT reducing the activation of such systems, voice-hearing may actually be eliminated, even if this were not the therapeutic goal. The effect of CFT on voice-hearing remains to be rigorously evaluated.

Finally, it is worth considering the specific types of voice-hearing to which this framework may be applicable. The framework we have set out focusses on voice-hearers who hear voices with a negative affective valence. However, what does this framework have to say about why 40% of people diagnosed with schizophrenia-spectrum disorders hear positive, supportive voices (Nayani and David, 1996)? How can it help us understand the voices heard by people who *only* hear positive, supportive voices, or people who began by hearing positive, supportive voices before their voices turned malevolent (McCarthy-Jones, 2017a)? Could it be that activation of the social-threat monitoring system could lead to negatively valenced voice-hearing, but that its opposing force, the compassion system, could also seed voice-hearing in the form of benevolent supportive voices? These could be internal versions of the secure base of attachment theory. Such questions remain to be elaborated. However, we suggest that not only can our proposed framework lead to the refinement of contemporary models of the causes and treatment of distressing voice-hearing, but that it can also provoke new questions to help us reconsider the nature of voice-hearing itself.

## Author Contributions

All authors listed have made a substantial, direct and intellectual contribution to the work, and approved it for publication.

## Conflict of Interest Statement

CH-M and PG are practitioners, supervisors and trainers of CFT and have received fees for providing these services. EL is also a trainer of CFT and has received fees for this. PG receives royalties from books he has published on CFT. The remaining author declares that the research was conducted in the absence of any commercial or financial relationships that could be construed as a potential conflict of interest.
